# Metabolic and transcriptome responses of RNAi-mediated *AMPKα* knockdown in *Tribolium castaneum*

**DOI:** 10.1186/s12864-020-07070-3

**Published:** 2020-09-23

**Authors:** Heng Jiang, Nan Zhang, Caihong Ji, Xiangkun Meng, Kun Qian, Yang Zheng, Jianjun Wang

**Affiliations:** 1 College of Horticulture and Plant Protection, Yangzhou University, Yangzhou, 225009 China; 2 Joint International Research Laboratory of Agriculture and Agri-Product Safety of the Ministry of Education, Yangzhou University, Yangzhou, 225009 China

**Keywords:** *Tribolium castaneum*, AMPK, RNA interference, Transcriptome, Lipid metabolism, Carbohydrate metabolism IIS pathway

## Abstract

**Background:**

The AMP-activated protein kinase (AMPK) is an intracellular fuel sensor for lipid and glucose metabolism. In addition to the short-term regulation of metabolic enzymes by phosphorylation, AMPK may also exert long-term effects on the transcription of downstream genes through the regulation of transcription factors and coactivators. In this study, RNA interference (RNAi) was conducted to investigate the effects of knockdown of *TcAMPKα* on lipid and carbohydrate metabolism in the red flour beetle, *Tribolium castaneum*, and the transcriptome profiles of dsTcAMPKα-injected and dsEGFP-injected beetles under normal conditions were compared by RNA-sequencing.

**Results:**

RNAi-mediated suppression of *TcAMPKα* increased whole-body triglyceride (TG) level and the ratio between glucose and trehalose, as was confirmed by in vivo treatment with the AMPK-activating compound, 5-Aminoimidazole-4-carboxamide1-β-D-ribofuranoside (AICAR). A total of 1184 differentially expressed genes (DEGs) were identified between dsTcAMPKα-injected and dsEGFP-injected beetles. These include genes involved in lipid and carbohydrate metabolism as well as insulin/insulin-like growth factor signaling (IIS). Real-time quantitative polymerase chain reaction analysis confirmed the differential expression of selected genes. Interestingly, metabolism-related transcription factors such as sterol regulatory element-binding protein 1 (SREBP1) and carbohydrate response element-binding protein (ChREBP) were also significantly upregulated in dsTcAMPKα-injected beetles.

**Conclusions:**

AMPK plays a critical role in the regulation of beetle metabolism. The findings of DEGs involved in lipid and carbohydrate metabolism provide valuable insight into the role of AMPK signaling in the transcriptional regulation of insect metabolism.

## Background

The survival of all organisms depends on the maintenance of energy homeostasis. AMP-activated protein kinase (AMPK) is a cellular energy sensor conserved across all eukaryotic species [13]. As a serine/threonine protein kinase complex, AMPK consists of a catalytic subunit α and two regulatory subunits, β and γ, and is activated in response to energy stress by sensing increases in ADP/ATP and AMP/ATP ratios, which leads to the activation of ATP-generating catabolic pathways including glycolysis and fatty acid oxidation and the inhibition of ATP-consuming anabolic pathways such as gluconeogenesis, fatty acid and protein synthesis [17]. While nucleotide-dependent phosphorylation of Thr172 in the α subunit by liver kinase B1 (LKB1) is the principal event required for full activation of AMPK in mammalian cells [19, 70], several studies have revealed the nucleotide-independent regulation of AMPK via the phosphorylation of Thr172 by calcium/calmodulin-dependent kinase kinase 2 in mammals (CAMKK2) [20, 23, 69].

Lipids and carbohydrates are major sources for energy storage and supply in cells. Under aerobic conditions, most energy is derived from fatty acids oxidation and the rest of energy is obtained from glucose oxidation. In contrast, glycolysis plays an important role in ATP production under anoxic condition [51]. Activation of AMPK occurs in response to stress circumstances such as starvation, exercise, hypoxia and ischemia, heat shock, and oxidative stress [9, 11, 18, 45, 47, 49, 67]. It is well known that AMPK can regulate lipid and carbohydrate homeostasis via direct phosphorylation of multiple downstream effectors. Acetyl-CoA carboxylase (ACC), the first rate-limiting enzyme in fatty acid synthesis, glycerol-3-phosphate acyltransferases (GPAT), the rate-limiting enzyme in triglyceride (TG) synthesis, and 3-hydroxy-3-methylglutaryl-CoA reductase (HMGR), the rate-limiting enzyme of cholesterol biosynthesis pathway, can be inhibited by AMPK through phosphorylation in rats [8, 42, 68]. Subsequently, AMPK was found to promote lipid absorption and release by directly phosphorylating lipases like hormone-sensitive lipase (HSL) and adipocyte-triglyceride lipase (ATGL) in mice and *Caenorhabditis elegans* [1, 44, 64]. On the other hand, AMPK also stimulates glycolysis via the phosphorylation and activation of 6-phosphofructo-2-kinase/fructose-2,6-biphosphatase 3 (PFKFB3) and 6-phosphofructo-2-kinase (PFK2) in human tissues [37, 38]. Additionally, AMPK activation was also shown to increase whole-body insulin sensitivity by phosphorylation of insulin receptor substrate-1 (IRS-1) in mouse [24] and insulin receptor (InR) in rodent muscle [10]. Given the functional attributes of AMPK in lipid and carbohydrate metabolism, AMPK is considered as an important therapeutic target for treating metabolic diseases including obesity and type 2 diabetes [43].

Although the role of AMPK in the regulation of cell metabolism is well studied in mammals, related research is still limited in insects. Notably, AMPK can regulate energy balance via modulation of transcriptional expression of metabolic enzymes in the long term, however, its downstream transcriptional pathways remains largely elusive [7]. Recently, we reported the transcriptional and post-translational activation of TcAMPKα by oxidative, heat and cold stresses in the red flour beetle, *Tribolium castaneum* [25]. In this study, RNAi was employed to determine the roles of *TcAMPKα* in lipid and carbohydrate metabolism. Comparison, annotation and classification of DEGs between dsTcAMPKα treatment and control groups were also conducted by high-throughput transcriptome sequencing to identify metabolism-related genes modulated by AMPK in *T. castaneum*.

## Results

### Effects of TcAMPKα suppression on TG, glucose and trehalose levels

**Fig. 1:**
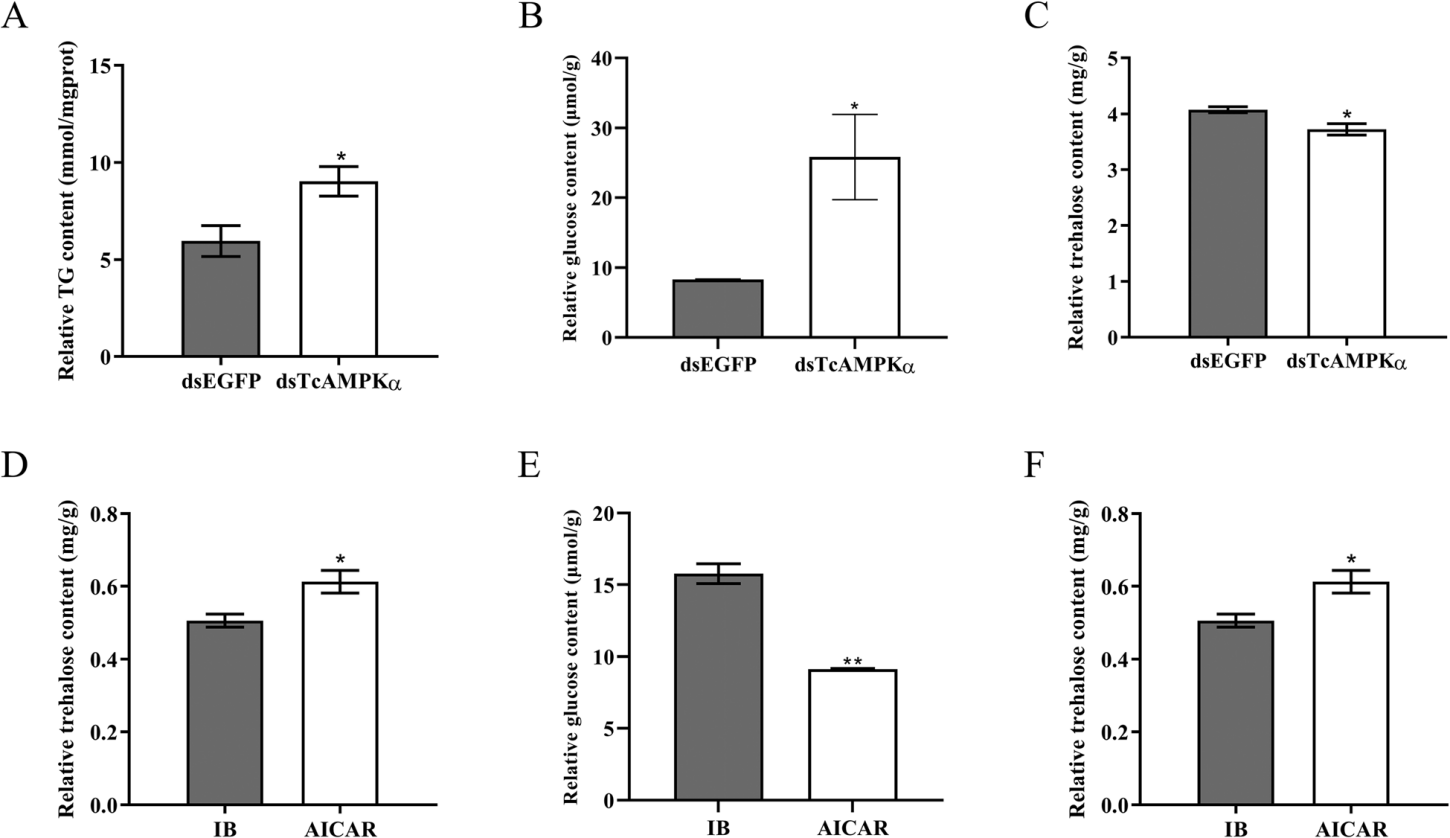
The change of TG, glucose and trehalose levels in dsTcAMPKα-treated and AICAR-treated *T. castaneum* larvae*.* **a**-**c**: Relative TG, glucose and trehalose levels of *T. castaneum* in dsTcAMPKα group compared with dsEGFP group. **d**-**f**: Relative TG, glucose and trehalose levels of *T. castaneum* in AICAR group compared with IB group. (by Student’s t-test, *p < 0.05, **p < 0.001)

RNAi was conducted to determine the effects of *TcAMPKα* knockdown on TG, glucose and trehalose levels. The injection of 20-day-old larvae with dsTcAMPKα reduced transcription levels by 95.50% ± 1.86% (ANOVA, df_2, 4_, F = 97.506, *P* value = 0.0027) on the sixth day after injection. TG measurement showed that the TG level in dsTcAMPKα group (9.03 ± 0.76 mmol/mgprot) was significantly increased by 53.49% ± 8.61% (ANOVA, df_1, 4_, F = 7.917, *P* value = 0.04813) when compared with the dsEGFP group (5.96 ± 0.78 mmol/mgprot) (Fig. 1a).

**Fig. 2:**
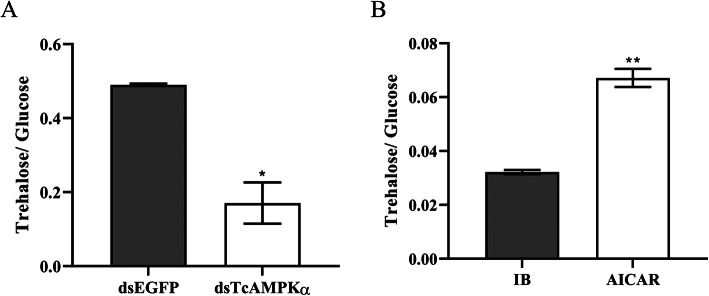
The ratio between trehalose and glucose under treatment with dsTcAMPKα and AICAR compared with control groups. (by Student’s t-test, *p < 0.05, **p < 0.001)

Similarly, increased glucose level by 62.34% ± 11.61% (ANOVA, df_1, 4_, F = 8.196, *P* value = 0.04580) was observed in the beetles injected with dsTcAMPKα (25.85 ± 6.12 μmol/g) when compared to the control beetles (8.32 ± 0.08 μmol/g) (Fig. 1b). However, the trehalose level in dsTcAMPKα group (3.73 ± 0.10 mg/g) was significantly reduced by 8.56% ± 3.01% (ANOVA, df_1, 4_, F = 9.357, *P* value = 0.0377) than that in dsEGFP group (4.08 ± 0.05 mg/g) (Fig. 1c). These data suggested that RNAi of TcAMPKα increased TG production and the ratio between glucose and trehalose (Fig. 2a).

### Effects of AICAR treatment on TG, glucose and trehalose levels

To confirm the RNAi results, the 20-day-old larvae were treated with an activator of AMPK 5-Aminoimidazole-4-carboxamide1-β-D-ribofuranoside (AICAR), and the TG, glucose and trehalose levels were measured. The results showed that the levels of TG and glucose in AICAR group were significantly decreased by 34.60% ± 5.74% (ANOVA, df_1, 4_, F = 10.770, *P* value = 0.03045) and 41.89% ± 2.27% (ANOVA, df_1, 4_, F = 93.320, P value = 0.0006), respectively, compared with injection buffer (IB) group, whereas the trehalose level increased by 17.07% ± 4.02% (ANOVA, df_1, 4_, F = 8.910, P value = 0.0405) in beetles treated with AICAR. These data suggested that activation of TcAMPK decreased TG production and the ratio between glucose and trehalose (Figs. 1 d-f; 2b).

### Transcriptome sequence and reads mapping

The dsTcAMPKα and dsEGFP groups were analyzed by RNA-Seq (three independent biological replicates of each treatment). A mean of 23,570,938 clean reads were generated among six independent libraries (T01-T06) (Table 1). Evaluation of clean data quality showed that the GC counts ranged from 42 to 45% and Q30 ratios were > 93%, indicating a high level of data quality. The alignment of clean reads to the reference genome database of *T. castaneum* showed that 83.50 and 77.15% reads of the dsEGFP and dsTcAMPKα groups were aligned on average, respectively (Table 2).

**Table 1:** Summary of the transcriptome sequencing data from the controls and dsTcAMPKα treated samples

Samples	ID	Clean Read Number	Clean Base Number	GC (%)	Q30 (%)
EGFP1	T01	21,746,430	6,523,929,000	42.79	93.39
EGFP2	T02	20,376,206	6,112,861,800	42.86	93.94
EGFP3	T03	26,557,829	7,967,348,700	43.85	92.88
dsTcAMPKα1	T04	24,192,194	7,257,658,200	44.91	93.25
dsTcAMPKα2	T05	23,215,801	6,964,740,300	45.01	92.85
dsTcAMPKα3	T06	25,337,170	7,601,151,000	45.54	92.75

**Table 2:** Summary of average read numbers based on the RNA-sequencing data

	dsTcAMPKα	dsEGFP
Total alignments	38,451,597	39,074,583
Reads aligned	37,340,338	37,891,202
Unique alignments	36,870,045	37,564,766
Not aligned	11,156,438	7,895,775

**Fig. 3:**
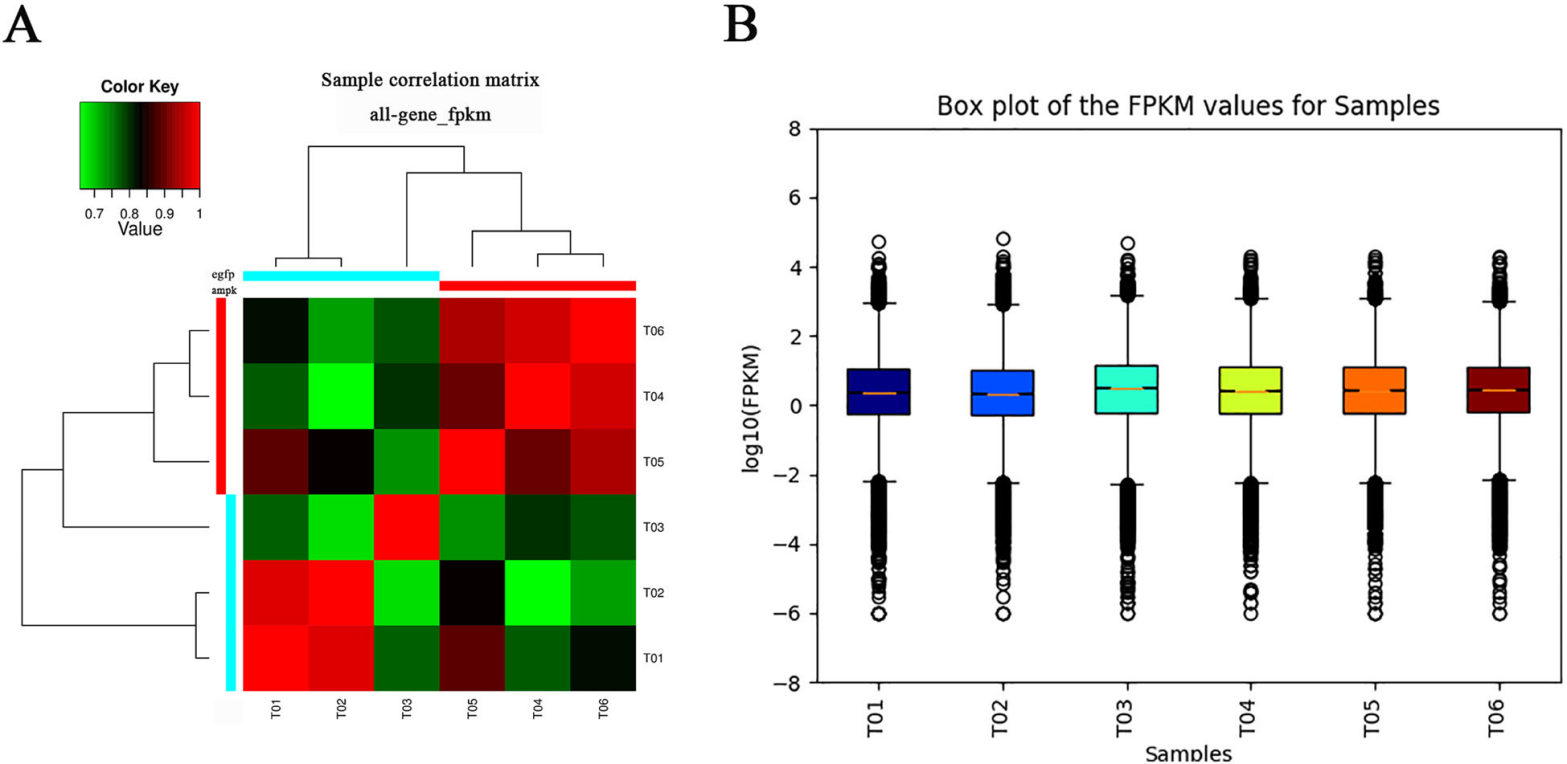
Correlation and box plot of the gene expression levels (FPKM) for all of the samples. A: Pearson correlations of gene expression levels of the six samples. The higher R value indicates closer relationship between two samples. B: Box plots of gene expression levels of the six samples. T01-T03 represent control sample (dsEGFP-injected group) libraries, and T04-T06 represent treatment sample (dsAMPKα-injected group) libraries

Correlation analysis was conducted with Pearson’s Correlation Coefficient (R value) to evaluate the biological repeatability based on expression values of each library [52]. The results showed that the Pearson’s Correlation Coefficient between the three control samples (T01-T03) was 0.81, and that of dsTcAMPKα treatment samples (T04-T06) was 0.94 (Fig. 3a). Box plot analysis revealed that the three samples in each group had similar expression distributions of reads, while the control and treatment groups had significantly different expression distributions (Fig. 3b).

Further annotation of expressed unigenes revealed that a total of 14,095 unigenes out of 31,944 unique sequences were annotated and classified into at least one database of Non-redundant (Nr), EuKaryotic Orthologous Groups (KOG), Clusters of Orthologous Groups of proteins (COG), Kyoto Encyclopedia of Genes and Genomes (KEGG), Protein family (Pfam), Gene Ontology (GO) and Swiss-Prot databases (Figure S1, Figure S2 and Table S2).

### Changes in gene expression profiles

**Fig. 4:**
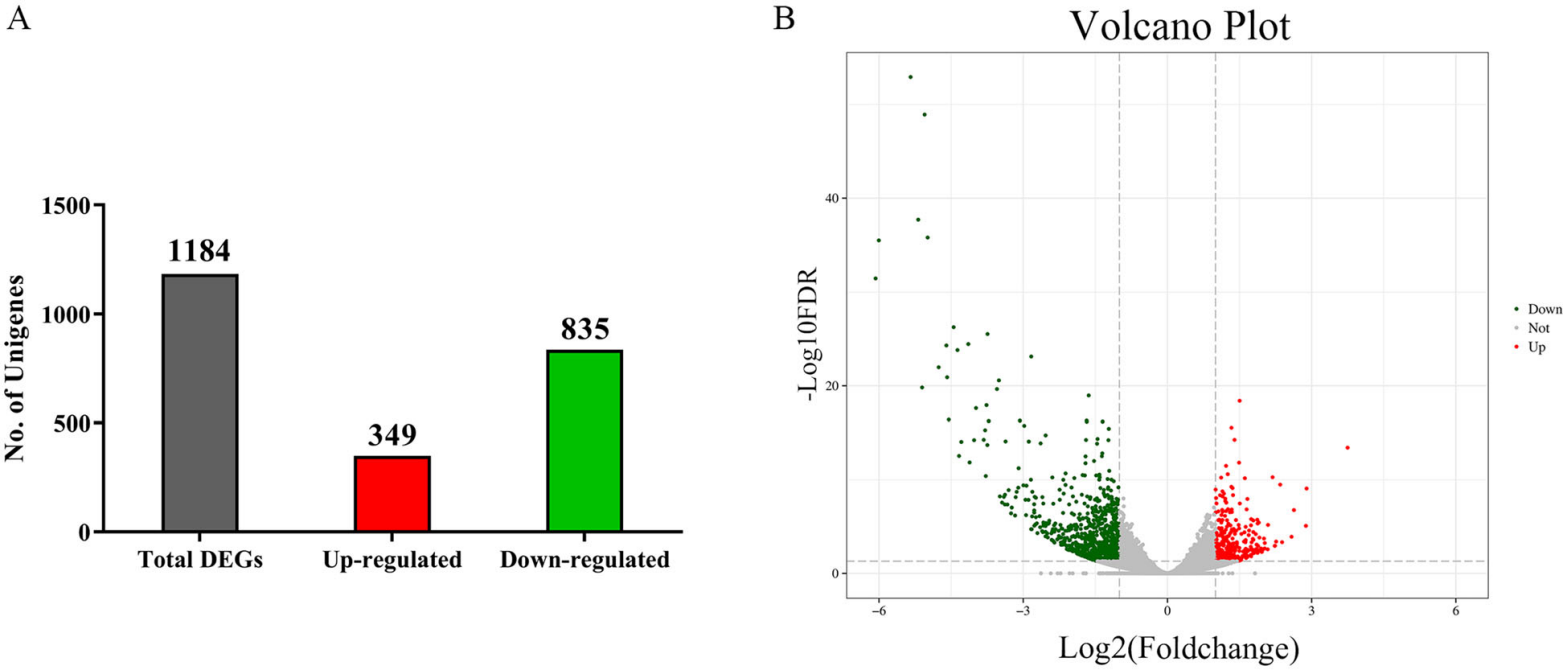
Differential gene expression analysis of *T. castaneum* in response to dsTcAMPKα treatment. A: Number of up- and down-regulated DEGs. B: Volcano plot of DEGs. X-axis: log_2_-fold change (treatment/control). Y-axis: -log_10_ (FDR). Red data points indicated up-regulated unigenes and green data points indicated down-regulated unigenes

To identify the effects of knock-down of *TcAMPKα* on global unigene expression patterns of *T. castaneum*, DEGs between dsTcAMPKα and dsEGFP groups were identified based on their Fragments Per Kilobase of transcript sequence per Million base pairs sequenced (FPKM) values. 1184 DEGs were obtained including 349 upregulated and 835 downregulated unigenes (Fig. 4a, b and Table S3). The log_2_-fold variation range of DEGs was between − 6.07 and 3.75 (Fold change from − 67.18 to 13.45, *P* value from 1.27 × 10^− 53^ to 0.04978).

DEGs were classified by searching against GO and KOG databases. GO term enrichments was used to further analyze physiological changes associated with DEGs in *T. castaneum*. GO enrichments revealed that the DEGs involved in cellular component category were enriched in cell part, cell, membrane and organelle (Figure S3). In molecular function category, most of the DEGs were enriched in binding and catalytic activity such as Retrovirus-related Pol polyprotein and ATPase inhibitor, while metabolic and cellular process were the most enriched subcategories in biological process (Figure S3).

**Fig. 5:**
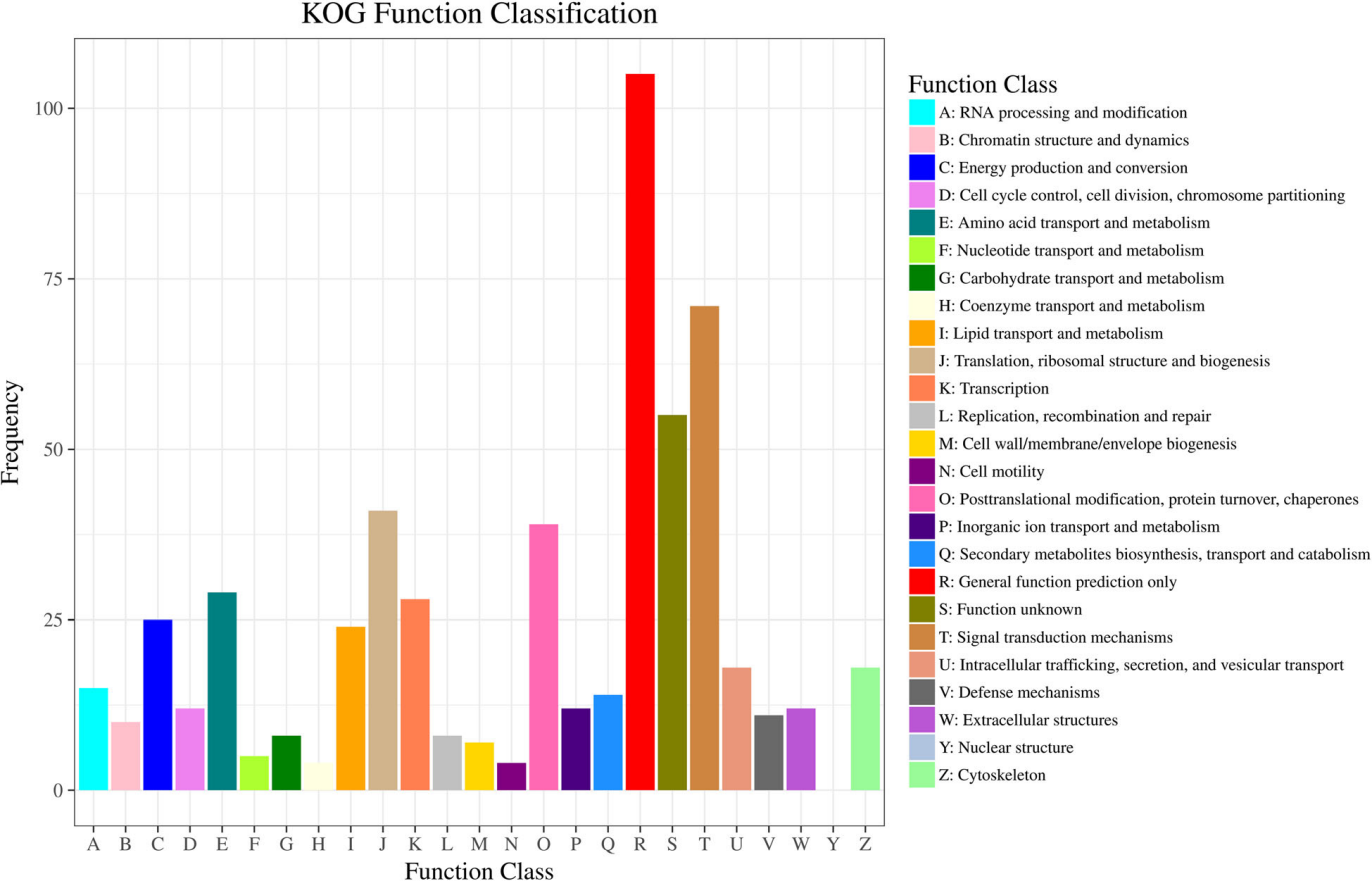
KOG function classifications of the differentially expressed unigenes. The X-axis represents names of 25 groups, and the Y-axis corresponds to the number of unigenes in the group

KOG database was used to annotate DEGs with specific physiological functions (Fig. 5, Table S4). Although the “General function prediction only” was the most abundant group among all subcategories in KOG database, a large number of DEGs were annotated into the metabolism classifications such as “Lipid transport and metabolism”, “Carbohydrate transport and metabolism”, “Amino acid transport and metabolism”, “Energy production and conversion” and “Secondary metabolites biosynthesis, transport and catabolism” (Table 3). Furthermore, most DEGs involved in protein translation were downregulated such as some ribosomal proteins (Table 3). Similarly, among the 42 DEGs in the post-translational modification class, 35 DEGs were down-regulated, including 3 heat shock proteins (Hsps), whereas the phosphatidylinositol 4,5-bisphosphate 3-kinase (PIK3) and InR2 involved in the signal transduction mechanisms were upregulated.

**Table 3:** DEGs involved in different functional categories of KOG database

Categories	Up-regulated DEGs		Down-regulated DEGs	
	NO.	Partial gene description	NO.	Partial gene description
Signal transduction mechanisms	26	tyrosine-protein phosphatase; phorbol ester/diacylglycerol-binding protein; lachesin; arf-GAP; citron Rho-interacting kinase; cyclic nucleotide-gated cation channel; E3 ubiquitin; sortilin; lipid transfer protein	23	neurogenic locus protein; extensin; SNF1A/AMP-activated protein kinase; cGMP-dependent phosphodiesterase; troponin C; sortilin; atrial natriuretic; sensory neuron membrane protein; regulator complex protein; tetra phosphatase
Translation, ribosomal structure and biogenesis	1	eukaryotic translation initiation factor	42	ribosomal protein; exosome complex component; ribonuclease; H/ACA ribonucleoprotein; eukaryotic translation initiation factor
Posttranslational modification, protein turnover, chaperones	7	papilin; heat shock protein 68a; pregnancy zone protein; fucosyltransferase; brain tumor protein; E3 ubiquitin;	35	mannosyltransferase; sulfotransferase; GILT-like protein; heat shock protein 23; ubiquitin; suppressor protein; collagenase; glutathione S-transferase; protein transport protein; NEDD8
Amino acid transport and metabolism	4	hydroxylase; protease; transporter; glucose dehydrogenase	30	proteinase; Carboxypeptidase; glucose dehydrogenase; trypsin
Lipid transport and metabolism	5	nose resistant to fluoxetine protein; fatty acyl-CoA reductase; apolipophorins; ATP-binding cassette; Fatty acid synthetase	17	dehydrogenase/reductase; NADPH; alpha-tocopherol; acyl-CoA-binding protein; nose resistant to fluoxetine protein; fatty acyl-CoA reductase; desaturase; monooxygenase
Energy production and conversion	1	titin isoform X4	23	cytochrome b-c1 complex; stunted; ATP synthase; V-type proton ATPase; NADH dehydrogenase; cytochrome c oxidase; cytochrome b5; acylphosphatase;
Secondary metabolites biosynthesis, transport and catabolism	5	fatty acyl-CoA reductase; cytochrome P450s; multidrug resistance-associated protein lethal	12	dehydrogenase/reductase; NADPH; laccase; fatty acyl-CoA reductase; cytochrome P450s
Carbohydrate transport and metabolism	1	mucin	6	amylase; chitinase; lactoylglutathione lyase; glycolipid transfer protein; myrosinase; peritrophic matrix protein

### Expression of genes involved in lipid metabolism, carbohydrate metabolism and insulin signaling

**Fig. 6:**
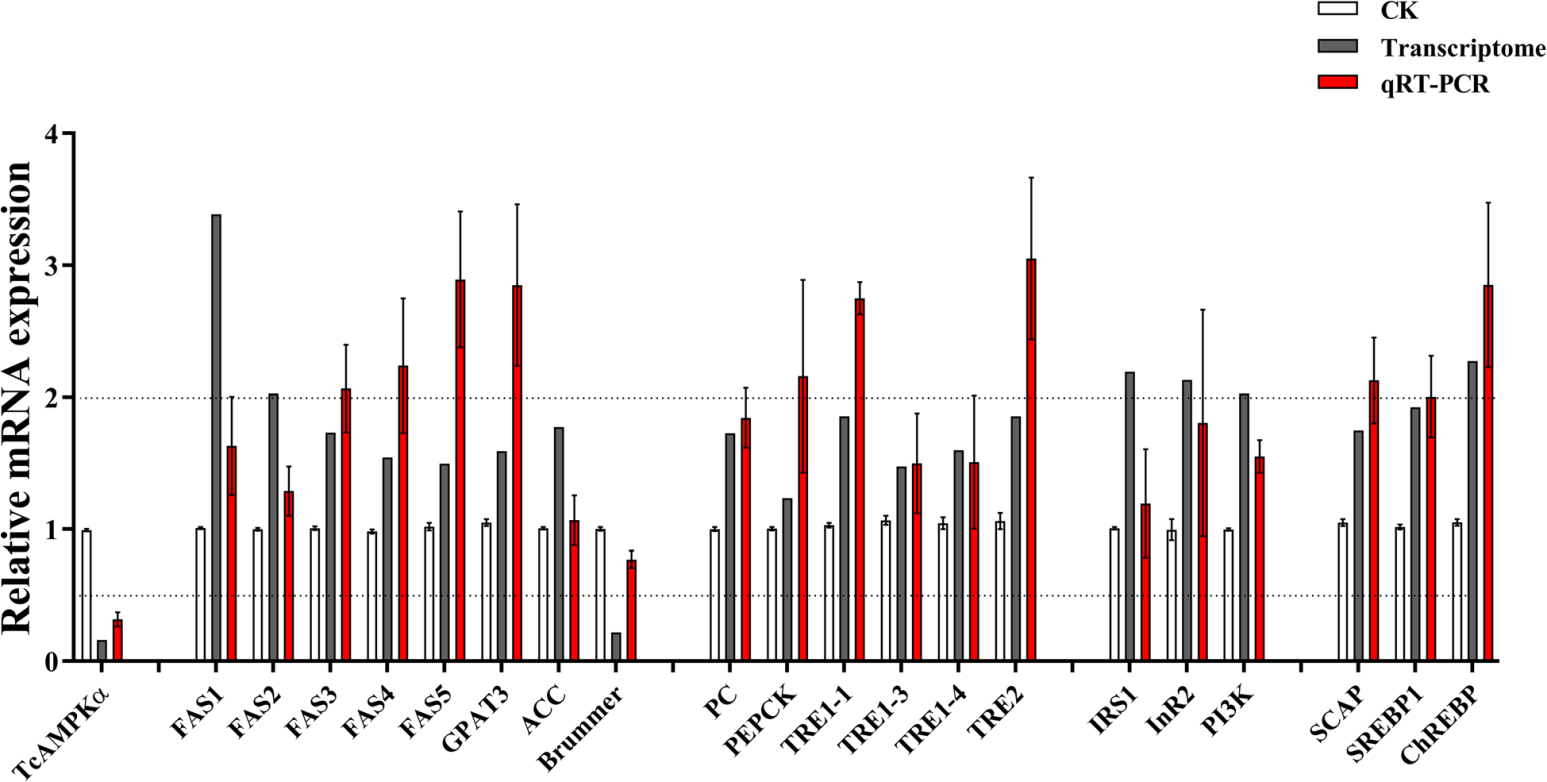
Comparison of gene expression patterns obtained by RNA-Seq and RT-qPCR

Besides the analysis of the entire gene set, we specifically checked for up- or downregulation of lipid and carbohydrate metabolism genes in our set of DEGs. The results showed that the insect adipose triacylglycerol lipase homologue, *brummer* (Accession no. TC011935), which is responsible for the first step of TG hydrolysis, was significantly downregulated with the expression log_2_-fold change of − 2.20 (Fold change: 0.22; ANOVA, df_1, 4_, F = 5.152, *P* value = 1.60 × 10^− 05^), whereas two fatty acid synthetase genes (FAS1–2) (Accession no. TC015337 and TC015340) (Fold change: 2.03 and 3.22, ANOVA, df_1, 4_, F = 9.182 and 157.306, *P* value = 0.03878 and 0.00023) involved in fatty acid biosynthetic pathways, and the transcription factor ChREBP (Accession no. TC010471) (Fold change: 2.27, ANOVA, df_1, 4_, F = 96.147, P value = 0.00061), a key regulator of glucose and lipid metabolism and fat storage [62], were upregulated with the expression log_2_-fold change from 1.02 to 1.76 (Fold change from 2.03 to 3.39) (Table 4). Knock-down of TcAMPKα also caused upregulation of genes involved in IIS pathway, including PI3K, IRS1 and InR2 (Accession no. TC011996, TC034013 and TC010784) (Fold change: 2.22, 2.19 and 2.46; ANOVA, df_1, 4_, F = 86.102, 14.636 and 34.155, P value = 0.00075, 0.01869 and 3.37 × 10^− 08^). To confirm the reliability of the DEG data, the expression levels of these DEGs were determined using RT-qPCR (Fig. 6 and Table 4). Gene expression levels validated by RT-qPCR showed the high consistency with transcriptome sequencing.

**Table 4:** DEGs encoding metabolism related proteins and transcription factors/ co-activators from *T. castaneum* responding to dsTcAMPKα treatment

Unigene name	padj	Description (blast)	Length (ORF bp)	Log_2_Ratio Transcriptome	Log_2_Ratio qRT-PCR
**Lipid metabolism**					
FAS1	0.038775298	fatty acid synthase	12,981	1.02	0.37
FAS2	0.056982009	fatty acid synthase	6522	1.76	0.71
FAS3	0.260213539	fatty acid synthase	7152	0.79	1.05
FAS4	0.425672932	fatty acid synthase	6630	0.63	1.16
FAS5	0.170696071	fatty acid synthase	6450	0.58	1.53
ACC	0.186316184	acetyl-CoA carboxylase	7005	0.83	0.10
GPAT3	0.046646995	glycerol-3-phosphate acyltransferases	1440	0.67	1.51
Brummer	1.60E-05	triacylglycerol lipase	1635	−2.20	−0.37
**Carbohydrate metabolism**					
TRE1–1	0.036902117	Trehalase1–1	1662	0.89	1.46
TRE1–3	0.450009964	Trehalase1–3	> 507	0.56	0.58
TRE1–4	0.007152475	Trehalase1–4	1812	0.68	0.59
TRE2	0.036902117	Trehalase2	1647	0.89	1.61
**Insulin signaling pathway**					
IRS1	0.018687502	insulin receptor substrate	2760	1.13	0.63
InR2	0.004419643	insulin-like receptor	4185	1.09	0.23
PI3K	3.37E-08	phosphatidylinositol 4,5-bisphosphate 3-kinase	3186	1.02	0.63
**Transcription factor and co-activator**					
SCAP	0.031742692	sterol regulatory element binding protein cleavage-activating protein	3783	0.81	1.12
SREBP1	0.036908768	sterol regulatory element binding protein 1	3078	0.94	1.74
ChREBP	0.000606393	carbohydrate response-element-binding protein	510	1.18	1.94

To be as inclusive as possible, less strict screening criteria (Fold change |log_2_(treatment/control) | > 0.5) were applied, and additional lipid and carbohydrate metabolism-related genes with the expression changed in transcriptome were selected for further verification using RT-qPCR. The results showed that five genes involved in fatty acid and triglyceride biosynthetic pathways including three FAS (FAS3–5) (Accession no. TC011522, TC015400 and TC000238) (Fold change: 1.73, 1.55 and 1.50; ANOVA, df_1, 4_, F = 1.717, 0.785 and 2.781, *P* value = 0.26021, 0.42567 and 0.17070), one ACC (Accession no. TC015612) (Fold change: 1.77; ANOVA, df_1, 4_, F = 2.539, P value = 0.18632), one glycerol-3-phosphate acyltransferases (GPAT) (Accession no. TC004512) (Fold change: 1.59; ANOVA, df_1, 4_, F = 8.092, *P* value = 0.04665), four trehalase (TRE) genes responsible for trehalose hydrolysis (Accession no. TC006698, LOC659620, TC004791 and TC006697) (Fold change: 1.86, 1.48, 1.60 and 1.86; ANOVA, df_1, 4_, F = 9.491, 0.699, 25.660 and 9.491, *P* value = 0.03690, 0.45001, 0.00715 and 0.03690), two genes involved in gluconeogenesis including pyruvate carboxylase (PC) (Accession no. TC032730) (Fold change: 1.73; ANOVA, df_1, 4_, F = 19.919, P value = 0.01113) and phosphoenolpyruvate carboxykinase (PEPCK) (Accession no. TC009072) (Fold change: 1.23; ANOVA, df_1, 4_, F = 1.491, P value = 0.28918) were significantly upregulated (Table 4). Interestingly, SREBP1(Accession no. TC007163) (Fold change: 1.93; ANOVA, df_1, 4_, F = 9.490, P value = 0.03691), the master regulator of lipid homeostasis, and SREBP cleavage-activating protein (SCAP) (Accession no. TC013456) (Fold change: 1.75; ANOVA, df_1, 4_, F = 10.483, P value = 0.03174), a central regulator of lipogenesis that controls the activity of SREBP [53] were also significantly upregulated (Table 4).

## Discussion

In insects, the energy for growth and development is mainly derived from the storage and utilization of lipids and carbohydrates in specific tissues such as fat body, midgut and oenocytes [39]. TG is the master form of lipids stored in fat body and plays an important role in energy storage and release [63]. Meanwhile, glucose (monosaccharide) and trehalose (disaccharide) provide energy through glycolysis [48]. Given that AMPK activates ATP-generating pathways and inhibits energy-consuming processes under conditions of low energy status [60], the attenuation of AMPK signaling in insects may disrupt the energy balance in vivo. In this study, we found that the TG and glucose levels in dsTcAMPKα-injected *T. castaneum* were significantly increased compared with dsEGFP-injected insects, while a decreased trehalose content was observed in dsTcAMPKα-injected insects. These results were further confirmed by in vivo AICAR treatment, which resulted in the decreased TG and glucose levels and increased trehalose content. Similarly, AICAR-induced AMPK activation resulted in significantly decreased TG level in lean and obese rodents, in vivo [6]. In human HepG2 cells, the kinase-inactive AMPKα increased lipid content and prevent the metformin from decreasing lipid accumulation [74]. However, lower TG levels were observed in *D. melanogaster* with reduced AMPK function during fed conditions [27]. On the other hand, while activation of AMPK triggered a reduction in glucose levels in vivo in mice [72], and increased hepatic glucose production was observed in AMPKα2 knockout mice [3], knockdown of hypothalamic AMPK activity in male Sprague-Dawley rats led to a significant suppression of glucose production [73]. These results indicate the complexity of the role of AMPK in the regulation of metabolic processes.

As an energy sensor that regulates cellular metabolism, AMPK not only has acute effects on metabolic enzymes by direct phosphorylation, but also shows long term action to change the transcriptional levels of metabolic proteins and enzymes. For example, activation of AMPK in liver and adipocytes can downregulate FAS activity and inhibit lipogenesis [36]. Activation of AMPK by 4-Hydroxyderricin and xanthoangelol downregulates *GPAT* in 3 T3-L1 cells [75], an enzyme necessary for triacylglycerol synthesis [66]. Similarly, in *C. elegans*, AAK-2 can inhibit fat synthesis under stress condition by downregulating lipid synthesis-related genes such as Δ9 fatty acid desaturases which can produce monounsaturated fatty acids to constitute TGs [55, 65]. To investigate the downstream transcriptional pathways of AMPK in *T. castaneum*, the transcriptomes of dsTcAMPKα-injected and dsEGFP-injected larvae under normal conditions were compared. Global gene expression profiles of dsTcAMPKα group were distinct from dsEGFP group with 1184 DEGs. Of particular interest is the upregulation of genes involved in fatty acid and TG biosynthetic pathways, such as FAS, ACC and GPAT, whereas the relative expression level of *brummer* involved in TG hydrolysis [14] was dramatically downregulated. We also observed the upregulation of several carbohydrate metabolism-related genes, such as TRE, and two key enzymes in gluconeogenesis (de novo synthesis of glucose), PC and PEPCK [16, 26], in the dsTcAMPKα-injected insects. The transcriptional changes of genes encoding metabolism enzymes might contribute to the increased TG and glucose levels and decreased trehalose content in dsEGFP-injected beetles.

AMPK activation has been reported to cause a reduction in transcriptional activity of several metabolism-related transcription factors. As a key transcription factor that regulates cellular lipogenesis in liver, skeletal muscle and adipose tissue, insulin-activated SREBP1 acts in synergy with glucose-senstive ChREBP, which mediates the response to dietary carbohydrates and is essential for regulating lipogenic gene expression [46]. AMPK-inhibited SREBP1 can block expression of some gluconeogenic and lipogenic genes, whereas SREBP1 overexpression can increase their transcription [12, 31, 54]. Activation of AMPK by metformin or an adenosine analogue suppresses the expression of SREBP1. In metformin-treated rats, hepatic expression of SREBP1 mRNAs and protein is reduced [76]. A recent study indicated that AMPK interacts with and directly phosphorylates SREBP1, suppresses SREBP1 cleavage and nuclear translocation, and represses SREBP1 target gene *Fas* expression in hepatocytes in response to metformin treatment, leading to reduced lipogenesis [34]. On the other hand, AMPK also phosphorylates Ser568 and reduce DNA binding capacity and promote nuclear exclusion of murine ChREBP [28, 29]. Interestingly, the promotion of PEPCK expression is associated with increased expression of SREBP-1 and ChREBP in high free fatty acid (HFFA)-treated hepatocytes [32], and a recent study also revealed the role of ChREBP in gluconeogenesis [58]. In this study, SREBP1, ChREBP and SCAP were significantly upregulated in dsTcAMPKα-injected insects, which in turn might modulated the expression of genes involved in lipid and carbohydrate metabolism.

IIS pathway was involved in the regulation of glucose and lipid metabolism (Saltie and Kahn 2001) [50]. In addition to the regulation of lipid synthesis, studies reveal expanding roles for SREBP1 in controlling pathways for insulin resistance [34], in which the pathological process involves a series of cascades, including defective activation of IRS and PI3K [22, 30, 59]. Overexpression of SREBP1 decreased *Irs-1* mRNA levels in a dose-dependent manner, and SREBP1 knockdown led to an upregulation of IRS-1 levels [34]. Further luciferase reporter assay confirmed that *Irs-1* promoter activity was repressed by SREBP1 overexpression [34]. However, in liver with Nonalcoholic fatty liver disease (NAFLD), IRS-1 expression was enhanced and correlated positively with SREBP1 expression. In contrast, IRS-2 expression decreased by 50% and was not correlated with SREBP1 [31]. In sebocytes, insulin-like growth factor-1 (IGF-1) induces SREBP-1 expressions at both mRNA and protein levels in a PI3K-dependent manner, accompanied by an increase in the transcription of SREBP-1 target genes such as FAS [57]. Additionally, insulin-stimulated endogenous ChREBP expression was also observed in HepG2 and primary hamster hepatocytes (Sirek et al. 2009) [56]. In this study, we observed the upregulation of IIS-related DEGs like IRS1, InR2 and PI3K in dsTcAMPKα-injected beetles, which might result in the upregulation of SREBP1 and ChREBP. Interestingly, it has been reported that knockdown in expression of insulin like peptide 2 (ILP2) caused a decrease in TRE mRNA levels in *T. castaneum* [71], suggesting the positive control of TRE transcription by IIS signaling. Further study is needed to clarify the mechanism of upregulation of IIS signaling in dsTcAMPKα-injected insects.

## Conclusion

This study confirmed that AMPK has an important role in the regulation of beetle metabolism. Specifically, our study showed that knockdown of AMPK causes alteration in expression levels of genes involved in lipid and carbohydrate metabolism as well as IIS signaling. Such investigations will help us understand the function of AMPK in transcriptional regulation of insect metabolism.

## Methods

### Experimental insects

The Georgia-1 (GA-1) strain of *T. castaneum* was reared at 30 °C and 50% relative humidity in 5% yeasted flour under standard conditions as described previously [15, 33].

### Double-strand RNA synthesis and injection

Gene specific primers (Table S1) with T7 promoter were designed to synthesize the dsRNAs targeting nucleotides 844–1285 (442 bp) of the ORF region of the *TcAMPKα* using TranscriptAid™ T7 High Yield Transcription Kit (Thermo Fisher Scientific, Waltham, MA, USA) according to the manufacturer’s instructions. The synthesized dsRNAs were diluted in diethyl pyrocarbonate (DEPC)-treated water with a concentration of 2 μg/μL, and about 200 ng of dsRNA in 200 nL IB was injected into 20-day-old larvae using a Nanoliter 2010 injector system (WPI, Sarasota, FL, USA) under a stereomicroscope. A total of 10 unsexed insect larvae were collected as a sample for RNA extraction at six- days post-injection. The dsEGFP-injected larvae (CK group) were set as controls in all injection experiments. Three biologically independent replicates were carried out with at least 100 insects (≥ 200 mg) in each replicate.

### Triglyceride (TG) measurement

Total TG levels were determined using the liquid TG (GPO-PAP) method [4]. Briefly, each replicate with 10 injected larvae were homogenized in 270 μL of PBS (0.1 mol/L PH 7.4) and centrifuged at 2500 rpm for 10 min. The supernatant was collected, and the TG level was analyzed by using TG Assay Kit (catalogue no. A110–1, Jiancheng Bioengineering Institute, Nanjing, China) according to the manufacturer’s instructions. Three independent biological replicates and four technical replicates were performed for every treatment.

### Glucose and trehalose measurement

Total glucose levels were measured using glucose content assay kit (catalogue no. BC2500, Solarbio Science & Technology, Beijing, China) according to the manufacturer’s instructions. Briefly, 10 larvae on day 6 after injection of dsTcAMPKα or dsEGFP were weighed and homogenized in 0.3 mL double distilled water. The supernatant was collected after centrifugation at 8000 g for 10 min and used to quantify the level of glucose at 505 nm with Multiskan FC microplate reader (Thermo Fisher Scientific, Waltham, MA, USA). Three independent biological replicates and 4 technical replicates were performed for every treatment.

Total trehalose level was measured using Trehalose content detection kit (catalogue no. A149–1-1, Jiancheng Bioengineering Institute, Nanjing, China) according to the manufacturer’s instructions. Six days after injection of dsTcAMPKα or dsEGFP, 10 larvae were homogenized in 0.3 mL extraction solution and incubated at room temperature for 45 min. The supernatant was collected after centrifugation at 8000 g for 10 min, and used to quantify the level of trehalose at 620 nm with Multiskan FC microplate reader (Thermo Fisher Scientific, Waltham, MA, USA). Three independent biological replicates and four technical replicates were performed for every treatment.

### AICAR treatment

To investigate the effects of AMPK activation on lipids and carbohydrates metabolism, 20-day-old larvae were injected with 200 nL of 1.6 mg/mL AICAR or IB as control. AMPK could be activated immediately by AICAR treatment among a couple of hours [40]. The total amounts of TG, glucose and glucose were determined at one- hour post-injection.

### RNA extraction, library construction and sequencing

Total RNA was isolated from 10 larvae of dsTcAMPKα or dsEGFP group on the sixth day after injection using TRIzol Reagent (Invitrogen, USA), and digested by RNase-free DNase I (Takara, Dalian, China) to remove genomic DNA contaminants. To ensure the quality of the samples for transcriptome sequencing, concentration and integrity of RNA samples were checked using a Nanodrop ND-1000 spectrophotometer (NanoDrop Technologies, Wilmington, DE) and an Agilent Bioanalyzer 2100 (Agilent, Palo Alto, CA), respectively. The qualified RNA samples were used for mRNA preparation and cDNA library construction. Three independent biological replicates were performed for every treatment. cDNA libraries were sequenced as described by Meng et al. (2019) [41].

### Sequence annotation

To ensure the accuracy of sequence alignment, raw reads were cleaned by removing adapter and primer sequences, reads with ambiguous nucleotides larger than 5% and low-quality reads. Clean reads were aligned to the reference genome of *T. castaneum* (Reference genome version: GCF_000002335.3_Tcas5.2_genomic.fna; Reference genome site: ftp://ftp.ncbi.nlm.nih.gov/genomes/all/GCF/000/002/335/GCF_000002335.3_Tcas5.2/GCF_000002335.3_Tcas5.2_genomic.fna.gz) [21] using HISAT2 (CCB, Johns Hopkins University, USA). Subsequently, the transcripts were subjected to blast against GO, KOG and KEGG databases. GO is an international standard classification system of gene function (Figure S1 and Table S2). KOG database is based on the phylogenetic relationship of bacteria, algae and eukaryotes (Table S2). The KEGG database provides a powerful tool to discover the pathways in which genes are involved (Figure S2). In addition, Nr, COG, Pfam, GO and Swiss-Prot databases were also used to annotate unigenes. The E-value threshold was set to 10^− 5^.

### Differentially expressed genes analysis

Transcript abundances were measured by FPKM [61]. Differential expression analysis of dsTcAMPKα and dsEGFP groups were performed using DESeq2 software [2]. Unigene with False Discovery Rate (FDR) values < 0.05 and fold change |log_2_(treatment/control) | > 1 were set as the threshold for DEGs. GO and KOG database annotations were further analyzed to understand the functions of DEGs in *T. castaneum*.

### Reverse transcription quantitative real-time polymerase chain reaction (RT-qPCR)

To validate the DEGs from the RNA-sequencing, RT-qPCR reactions were performed on the Bio-Rad CFX 96 Real-time PCR system using TB Green™ Premix Ex Taq™ (Takara, Dalian, China) and gene specific primers (Table S1). The stably expressed gene encoding ribosomal protein S3 (rps3, GenBank: CB335975) was used as a reference gene [5]. PCR conditions were set as an initial incubation of 95 °C for 30s, 40 cycles of 95 °C for 5 s and 60 °C for 30s, and a final melting curve analysis was performed. The mRNA levels were normalized to reference gene with the 2^-ΔΔCT^ method Livak and Schmittgen [35]. The means and standard errors (mean ± SE) for each time point were obtained from the average of at least three biologically independent sample sets.

### Statistical analysis

Statistical analysis was performed using Graphpad Prism 6 (GraphPad Software Inc., San Diego, USA) by one-way analysis of variance, followed by Tukey’s Honestly Significant Difference test. All data are presented as the mean ± SE.

## Supplementary information

**Additional file 1.**

**Additional file 2.**

**Additional file 3.**

**Additional file 4.**

**Additional file 5.**

**Additional file 6.**

**Additional file 7.**

## Data Availability

All data generated or analyzed during this study are included in this article and its supplementary information files. We have uploaded the raw data of our transcriptome sequencing to GEO database. The GEO accession number is GSE155634.

## Abbreviations

ACC: acetyl-CoA carboxylase
AICAR: 5-Aminoimidazole-4-carboxamide1-β-D-ribofuranoside
ATGL: adipocyte-triglyceride lipase
AMPK: AMP-activated protein kinase
CAMKK2: calcium/calmodulin-dependent kinase kinase 2
CHREBP: carbohydrate response element-binding protein
COG: Clusters of Orthologous Groups of proteins
DEGs: differentially expressed genes
DEPC: diethyl pyrocarbonate
FAS: fatty acid synthetase
FDR: False Discovery Rate
FPKM: Fragments Per Kilobase of transcript sequence per Million base pairs sequenced
GO: Gene Ontology
GPAT: glycerol-3-phosphate acyltransferases
HMGR: 3-hydroxy-3-methylglutaryl-CoA reductase
HSL: hormone-sensitive lipase
Hsps: heat shock proteins
IB: injection buffer
IIS: insulin/insulin-like growth factor signaling
ILP2: insulin like peptide 2
InR: insulin receptor
IRS-1: insulin receptor substrate-1
KEGG: Kyoto Encyclopedia of Genes and Genomes
KOG: EuKaryotic Orthologous Groups
LKB1: liver kinase B1
NAFLD: Nonalcoholic fatty liver disease
Nr: Non-redundant
PC: pyruvate carboxylase
PEPCK: phosphoenolpyruvate carboxykinase
Pfam: Protein family
PFK2: 6-phosphofructo-2-kinase
PFKFB3: 6-phosphofructo-2-kinase/fructose-2,6-biphosphatase 3
PIK3: phosphatidylinositol 4,5-bisphosphate 3-kinase
RNAi: RNA interference
SCAP: SREBP cleavage-activating protein
SREBP1: sterol regulatory element-binding protein 1
TG: triglyceride
TRE: trehalase
