# Supplementary material for: Metabolic and transcriptome responses of RNAi-mediated AMPKα knockdown in Tribolium castaneum
Source: BMC Genomics. 2020 Sep 23;21:655. doi: 10.1186/s12864-020-07070-3 (PMC7510082; doi:10.1186/s12864-020-07070-3)
Supplement: Supplementary file 1 — Additional file 1. [file 12864_2020_7070_MOESM1_ESM.docx]

**Table S1.** Oligonucleotide primers used for RT-PCR and RT-qPCR

| Primer name | Sequence (5′ to 3′)Primer name | Description |
| --- | --- | --- |
| TcAMPKαi-F1  TcAMPKαi-R1 | GTTCGGGGGTTGATTACTGCC | TcAMPKα dsRNA |
|  | GGACTTCCTTCTCCTGCACCC |  |
| dsEGFP-F  dsEGFP-R | CCTCGTGACCACCCTGACCTAC | EGFP dsRNA |
|  | CACCTTGATGCCGTTCTTCTGC |  |
| TcAMPKα-F4  TcAMPKα-R4 | ACATCCTTGGCCAGACTTTG | TcAMPKα RT-qPCR |
|  | GCCGAAACAGCTTCAAGTTC |  |
| Tcrps3-F1  Tcrps3-R1 | ACCGTCGTATTCGTGAATTGAC | rps3 RT-qPCR |
|  | ACCTCGATACACCATAGCAAGC |  |
| FAS1 qF  FAS1 qR | AATTCCTCCAACTGGCGGCTTC | TcFAS1 RT-qPCR |
|  | ATCGCCTCCAGACTAACCTCCA |  |
| FAS2 qF  FAS2 qR | GGACCACGTAATTGTAGAGTCTTGC | TcFAS2 RT-qPCR |
|  | TCCCTGACTCGGCAATTAAACATC |  |
| FAS3 qF  FAS3 qR | GCCACCACGTAAACGCAATCAG | TcFAS3 RT-qPCR |
|  | ATCGTCGTGCATCGTCGGTTAC |  |
| FAS4 qF  FAS4 qR | AACCCAACCACCAGTTCAGC | TcFAS4 RT-qPCR |
|  | TCGCTCACCATCTCCACCTT |  |
| FAS5 qF  FAS5 qR | TCATGGCTTCGTCTCTCGGA | TcFAS5 RT-qPCR |
|  | GGACACCCTGTTTGGCATCA |  |
| ACC qF  ACC qR | CTCACTTGTTTGCATGGACCACTAG | TcACC RT-qPCR |
|  | CCCAAATTTCCCACTGGCCTAAT |  |
| *bmm* qF  *bmm* qR | AAAGGTCACCGCTTCAGAAAGTACC | Tc*bmm* RT-qPCR |
|  | ATTTCCCGTCGTCGTCGTAATGC |  |
| GPAT3 qF  GPAT3 qR | TGAGTACGGTCGTGCAAACA | TcGPAT3 RT-qPCR |
|  | GGGCAGTGATTTCATCGTCG |  |
| InR2 qF  InR2 qR | AAATACACTACCCGTTCGGATTCG | TcInR RT-qPCR |
|  | TTCAACGCAGCTTATACACGCTAC |  |
| PI3K qF  PI3K qR | CCTACTGGACATCAAAGCGGACTT | TcPI3K RT-qPCR |
|  | GCAGGTGAAGACATACGAGGACAT |  |
| IRS1 qF  IRS1 qR | ACACATGACCGACCTCGTCAAGAT | TcIRS1 RT-qPCR |
|  | GCCCGACATCATCAACGCACAT |  |
| TRE1-1 qF | CGATCCAAATGGCCCGACTT | TcTRE1-1 RT-qPCR |
| TRE1-1 qR | GTCAGTGTCCTGGAGCAACT |  |
| TRE1-3 qF | TCATTCGTCACAAGCGAGCC | TcTRE1-3 RT-qPCR |
| TRE1-3 qR | ATTCGCGCCGTTTGGACCAT |  |
| TRE1-4 qF | TTCTCGCCGAGTCGAGTCAT | TcTRE1-4 RT-qPCR |
| TRE1-4 qR | CATTTGCACCGTGTGGAGGA |  |
| TRE2 qF | GCCAGGGTTGTAGCACTGAT | TcTRE2 RT-qPCR |
| TRE2 qR | CCACTACACTGAACCAGCGA |  |
| PC qF | GGACAGAGCCCACATTCACA | TcPC RT-qPCR |
| PC qR | TACAGCGTCAATGCCGTTGT |  |
| PEPCK qF | ACCGAAGTTGGACGCTTTGC | TcPEPCK RT-qPCR |
| PEPCK qR | CACCACGCTGATGCCTTTCA |  |
| SCAP qF | TTGGTCAGTGCCTCGCTAGA | TcSCAP RT-qPCR |
| SCAP qR | ACGCCTTCCTGTCCACATTG |  |
| SREBP1 qF | AGCTGTTCCCAGAAGATGCC | TcSREBP1 RT-qPCR |
| SREBP1 qR | TGCTGCTTGAGAGGTGAGAG |  |
| ChREBP qF | AGCGATGCTTCAGAAGGGTG | TcChREBP RT-qPCR |
| ChREBP qR | CTGCTGCCTCAAACACTCCA |  |
