# Supplementary figures and images for: Metabolic and transcriptome responses of RNAi-mediated AMPKα knockdown in Tribolium castaneum
Source: BMC Genomics. 2020 Sep 23;21:655. doi: 10.1186/s12864-020-07070-3 (PMC7510082; doi:10.1186/s12864-020-07070-3)

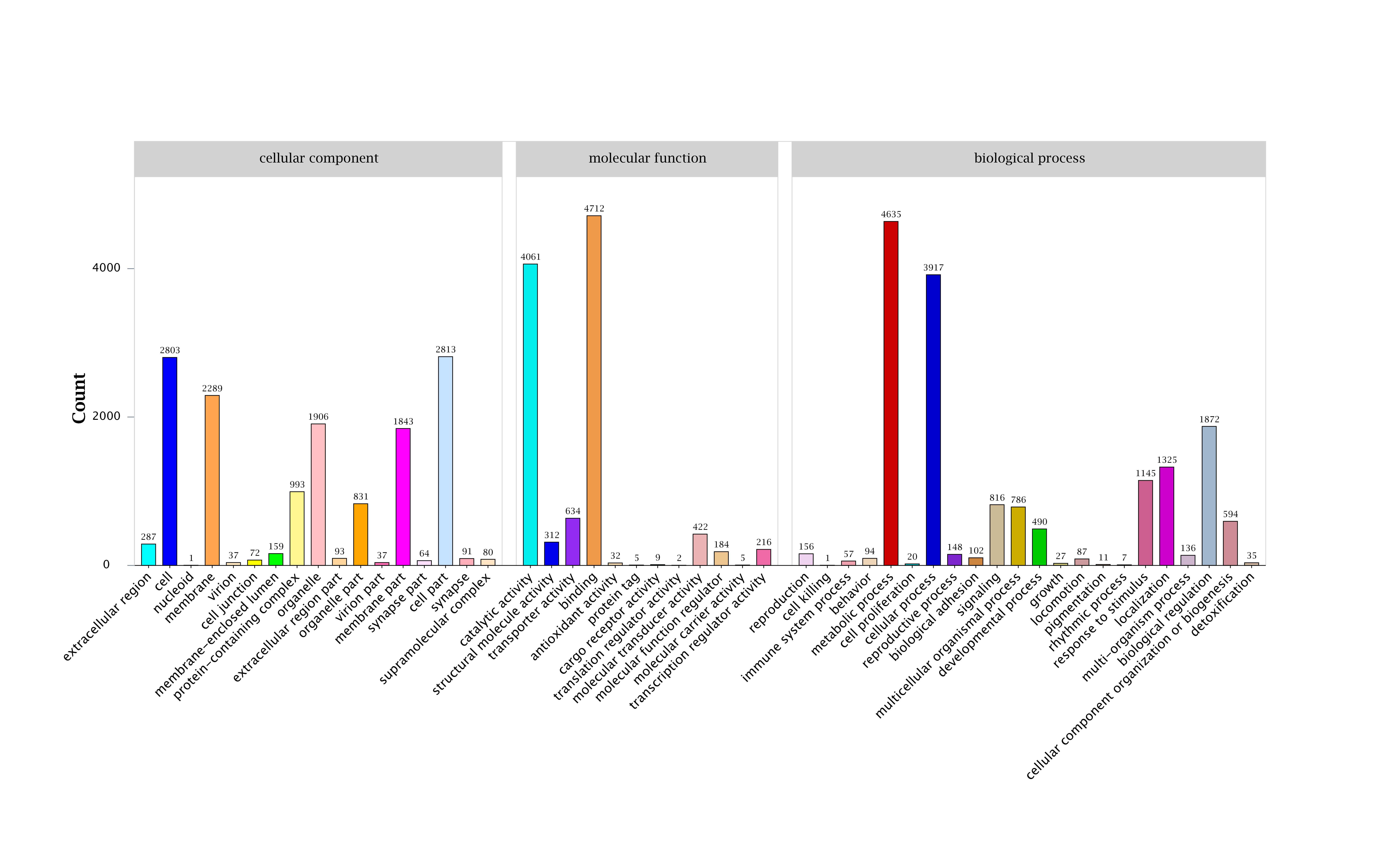

Supplement: Supplementary file 5 — Additional file 5. [file 12864_2020_7070_MOESM5_ESM.jpg]

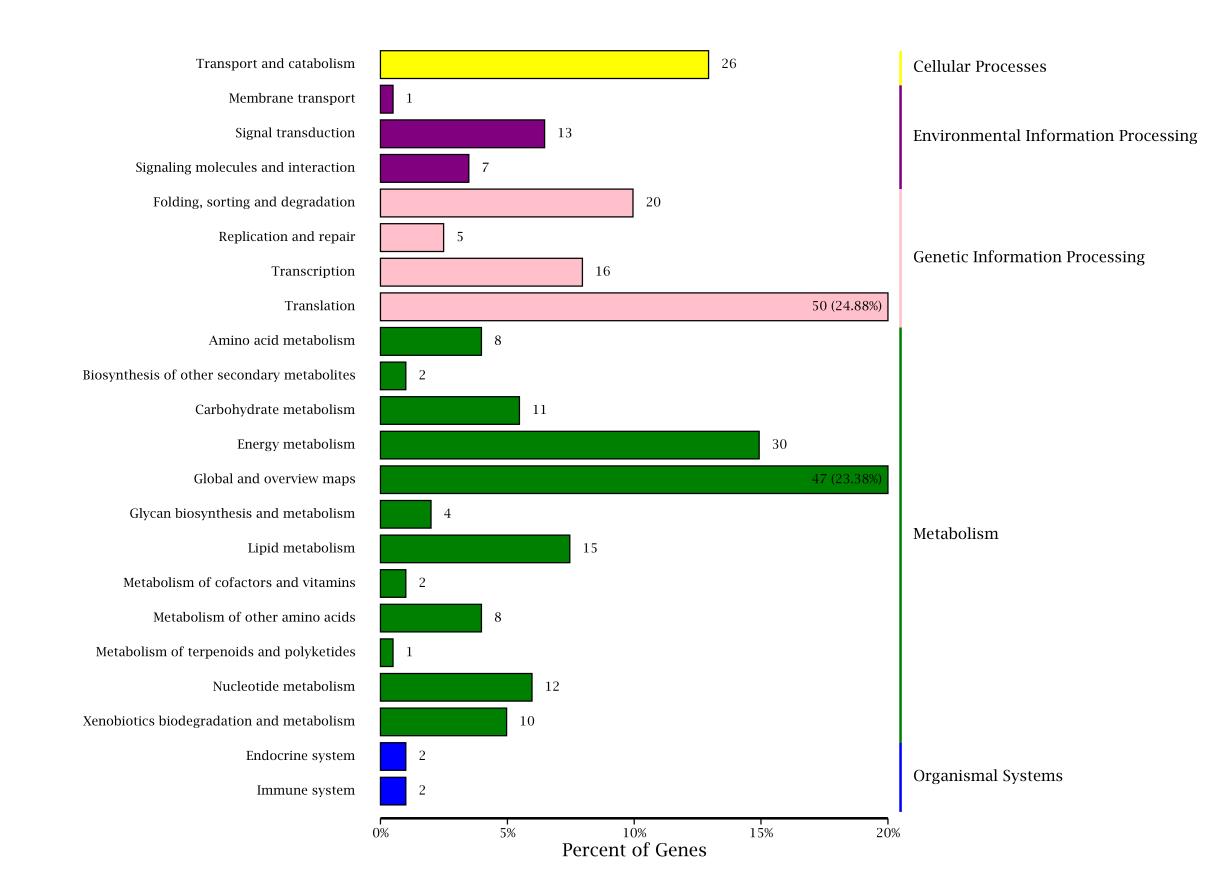

Supplement: Supplementary file 6 — Additional file 6. [file 12864_2020_7070_MOESM6_ESM.jpg]

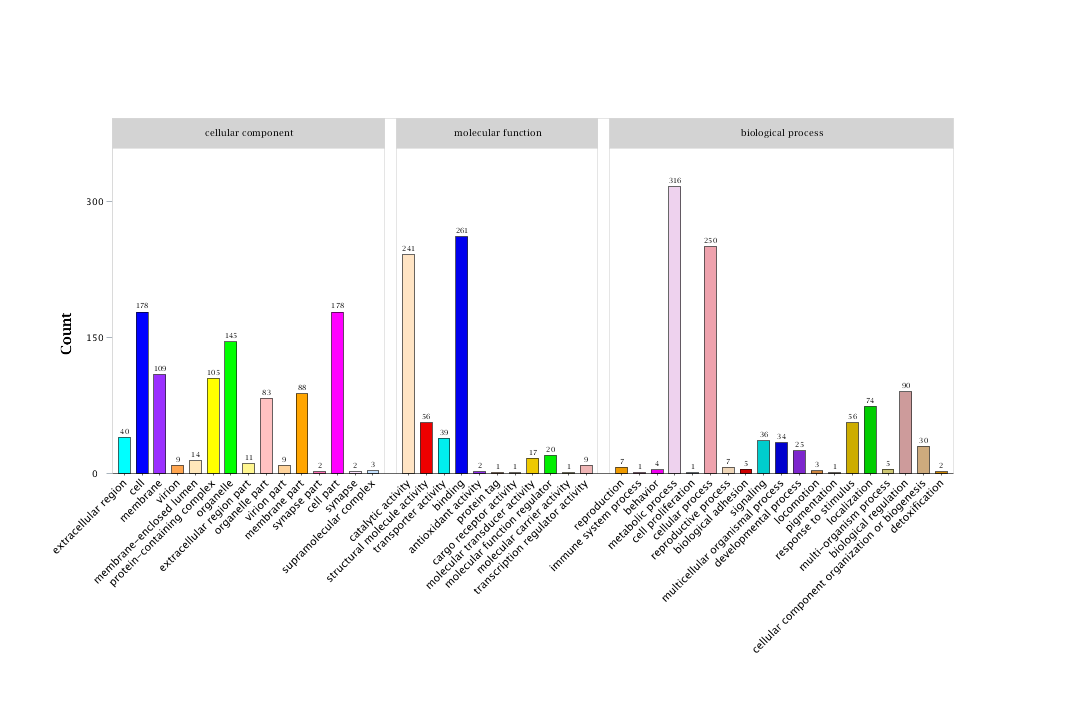

Supplement: Supplementary file 7 — Additional file 7. [file 12864_2020_7070_MOESM7_ESM.png]
